# Hyperbranched poly(ϵ-lysine) substrate presenting the laminin sequence YIGSR induces the formation of spheroids in adult bone marrow stem cells

**DOI:** 10.1371/journal.pone.0187182

**Published:** 2017-12-12

**Authors:** Valeria Perugini, Steve T. Meikle, Anna L. Guildford, Matteo Santin

**Affiliations:** Centre for Regenerative Medicine and Devices, School of Pharmacy and Biomolecular Sciences, University of Brighton, Brighton, United Kingdom; University Hospital Modena and Reggio Emilia, ITALY

## Abstract

Unlike the fibroblast-like cells formed upon monolayer culture of human mesenchymal stem cells, the natural stem cell niche of the bone marrow and other types of tissues favours the formation of 3-dimensional (3D) cell clusters. The structuring and biological activity of these clusters are regulated by the contacts established by cells with both the basement membrane and neighbour cells and results in their asymmetric division and the consequent maintenance of both a stem population and a committed progeny. The present work demonstrates the potential of a synthetic substrate to mimic the stem cell niche *in vitro*. The side amino groups of a linear Poly-L-lysine were modified with hyperbranched poly-(ϵ-lysine) peptides, named as dendrons, tethered with the laminin-mimicking sequence, YIGSR. These dendrons presented the YIGSR sequence at the uppermost molecular branching ensuring a controlled spacing of the bioligand. When used to coat the surface of tissue culture plates in a serum-free *in vitro* cell culture system, the substrate was able to mimic the most relevant features of the basement membrane of the stem cell niche, i.e. the mesh structure of Collagen Type IV and the availability of laminin bioligands relevant to integrin biorecognition. The substrate biomimetic properties were tested for their ability to support the formation of human bone marrow mesenchymal stem cells (hMSCs) 3D spheroids similar to those observed in the natural stem cell niches and their ability to maintain stem cell pluripotency markers. These features were related to the substrate-specific expression and localisation of (i) cell adhesion receptors (i.e. β-integrin and N-cadherin), (ii) transcription factors of pluripotency markers and cytoskeleton protein and (iii) regulators of cell migration throughout cell culture passages 2 to 4. The results clearly demonstrate the formation of 3D spheroids starting from the asymmetric division of substrate-adhering spread cells, the clustering of relevant integrins and the expression of specific intracellular pathways controlling cytoskeleton formation suggesting their potential use as a substrate for the handling of stem cells prior to transplantation procedures.

## Introduction

Testing the quality of bone marrow-derived human stem cells (hMSCs) and performing an *in vitro* expansion is widely considered a key pre-clinical step for any reliable cell-based treatment [1]. To this end, it is important to prevent the uncontrolled loss of the multipotent phenotype that takes place in standard culturing conditions. Concerns linked to the culturing of hMSCs in serum-enriched media and/or following supplementation with growth factors from animal sources [2, 3] have partially been overcome by the development of serum-free media [4]. However, there is still a demand for substrates capable of preventing the uncontrolled differentiation of hMSCs into fibroblast-like cells. Substrate alternatives to tissue culture plate (TCP) have been made available, but they still lead to the formation of fibroblast-like cells or they direct the stem cell multipotent phenotype towards specific cell differentiation pathways [5, 6, 7]. For example, poly-L-lysine (PolyK) substrates have been shown to partly direct stem cells towards a neural phenotype [8]. Such a differentiation was shown to increase when PolyK was modified with specific bio-active molecules such as the laminin-mimicking peptide sequence (i.e. YIGSR) [9].

As far as the maintenance of the hMSC multipotent phenotype is concerned, it is widely accepted that stem cell culture would be better performed on substrates that can mimic the microenvironment of the natural stem cell niche [10]. However, many studies have reported that hMSCs within their niche are regulated by a variety of signals, which are hard to recapitulate in culture [11]. Recently, a method to produce and stabilise an instructive stem cell niche has been obtained through the culturing of hMSC on fibronectin-coated glass substrates and subsequent de-cellularisation of the secreted matrix [12]. Although, this method can be considered a significant step forward in the *in vitr*o handling of stem cells, its use is still limited by the lack of stability and batch-to-batch variations of the natural substrate as well as the formation of typical colony forming units with morphological features different from those found in the natural stem cell niche of most tissues [13]. Recent studies have shown that cells within a typical bone marrow peri-vascular stem cell niche are organised as 3D “mesenspheres” rather than as 2D colonies of spread cells [14].

A constant feature of the stem cell niche is the basement membrane (BM) that is characterised by the mesh-like structure of collagen type IV and by the presence of proteins like laminin playing an important role in cell recognition processes. Through these components, the BM supports hMSC anchorage and asymmetric division, a process that ensures the maintenance of both a stem cell population and a progeny [15]. More specifically, it is believed that this asymmetric division of hMSCs within the niche is primarily controlled by the ability of the components of the BM to control the clustering of relevant stem cell receptors, the integrins [16].

In this paper, the mimicking of the main features of the BM in hMSC *in vitro* culturing was pursued through the use of a substrate coating based on a linear poly-L-lysine (PolyK) the side amino groups of which were modified with a hyperbranched poly(ϵ-lysine) peptides, called dendrons, which exposed at their uppermost third branching generation (Gen3) the laminin sequence YIGSR. When grafted to the side chains of PolyK, these dendrons [CGen3K(YIGSR)_16_] contributed to mimic the BM mesh-like structure of collagen Type IV while enabling the nanometric control of the laminin YIGSR presentation to the cell integrins. The behavior of adhering and proliferating cells was compared to that usually obtained upon culturing on TCP and non-modified PolyK where typical fibroblast-like colonies were formed. The study unveiled the ability of these substrates to induce the organisation of hMSCs into spheroids similar to those present in the natural stem cell niche through integrin clustering and the maintenance of pluripotency through the high transcriptional activity of relevant markers, thus suggesting their potential use in standardised hMSC culturing.

## Materials and methods

### Dendron synthesis and characterisation

**Fig 1:**
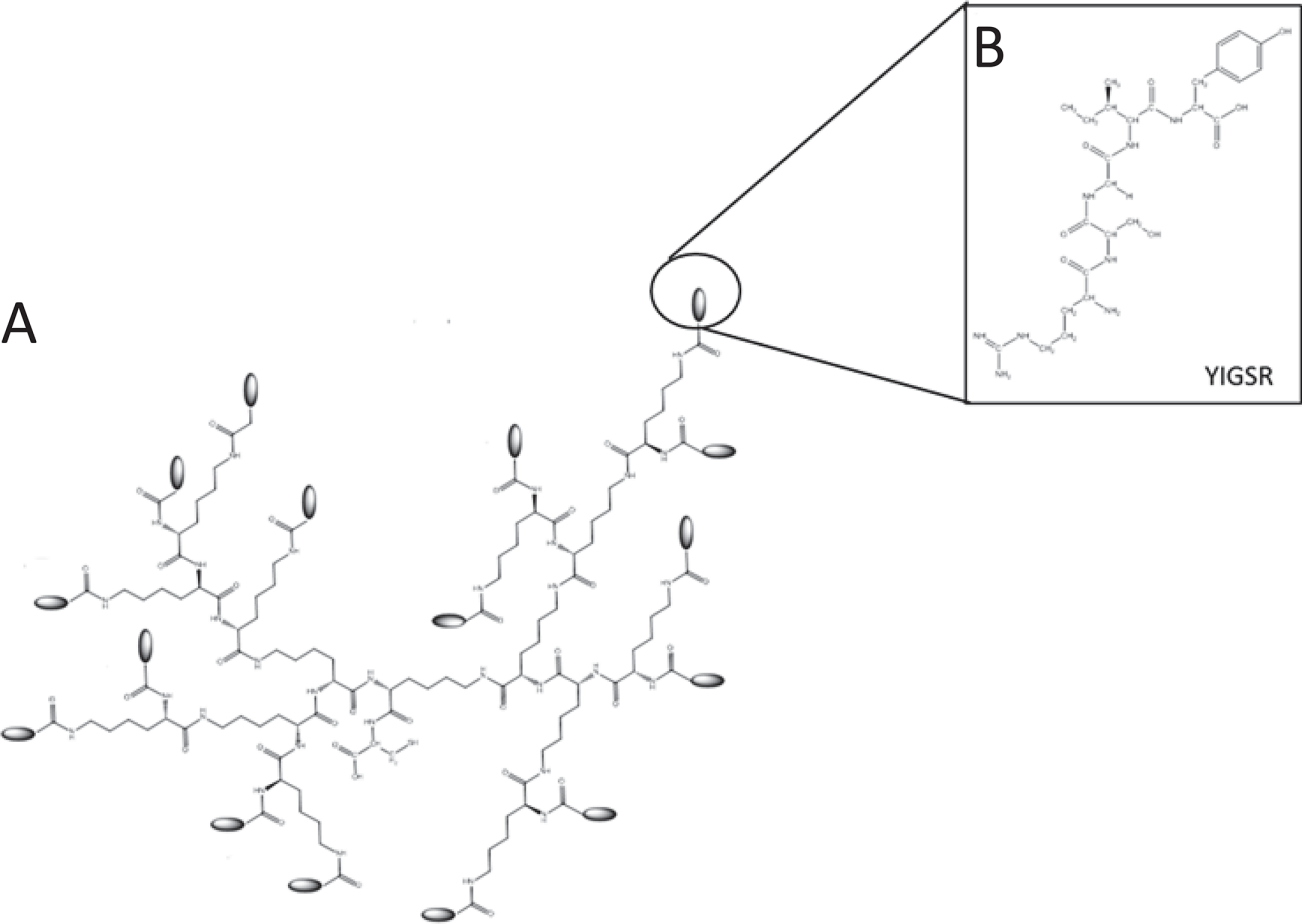
Chemical structure of a CGen3K(YIGSR)_16_. The dendron (A) was designated to expose sixteen YIGSR (B) on their uppermost branching generation.

Hyperbranched poly(ϵ-lysine) dendrons (K) with a cysteine core (C) and laminin linear peptide sequences (YIGSR) exposed on their second and third branching generation (Gen3) were assembled using Fmoc solid phase peptide method by a microwave synthesiser (Biotage Initiator) (Fig 1). Upon completion of their synthesis, the dendrons were cleaved using 95% v/v trifluoroacetic acid (TFA, Fisher Scientific), 2.5% v/v deionised H_2_O and 2.5% v/v triisopropylsilane (TIS, Sigma-Aldrich) and analysed by mass spectrometry (MS), HPLC and TLC for purification and synthesis analysis as described by Meikle *et al* [17].

### Synthesis of CGen3K(YIGSR)_16_-modified PolyK

The functionalisation of recombinant PolyK (Mw 70,000–150,000, Sigma-Aldrich) with CGen3K(YIGSR)_16_ was performed by initially dissolving dendrons in a 2-(N- morpholino)ethanesulfonic acid (MES, Sigma-Aldrich, UK) (0.1 M) buffer solution, pH 6.5. CGen3K(YIGSR)_16_ were then covalently bound to the free PolyK at a final concentration of 0.1% w/v through activation of their carboxyl groups with 10 mM N-hydroxysulfossucinimide (sulfo-NHS) and 4 mM 1-ethyl-3-(dimethylaminopropyl) carbodiimide (EDC, Sigma-Aldrich, UK) for 1 hour, room temperature. PolyK modification was performed under continuous stirring overnight, room temperature. Afterwards, the modified polymer was wash-filtered through Vivaspin® Turbo 4, 5000 MWCO, PES ultrafiltration membrane centrifuge tubes (Fisher Scientific, UK) to remove any impurities.

### Coating of tissue culture polystyrene plate surfaces

On completion of the functionalisation process, 150 μL of either non-modified or modified 0.1% w/v PolyK in ethanol/water (75:25 v/v) solution were used to coat the surface of 24-well TCP. The solution was left to react for 1 hour at room temperature and then to air dry overnight. The treated-wells were rinsed thoroughly with distilled water and sterilised by a 256 nm wavelength UV lamp (Perkins) in a sterile tissue culture hood for 1 hour.

### Characterisation and quantification of coating substrates

The stability of non- and modified PolyK coatings was assessed by a colorimetric bicinchoninic acid (BCA, Sigma-Aldrich, UK) assay. BCA reagent was mixed with copper (II) sulphate-pentahydrate solution at the final ratio 1:50 under continuous mechanical stirring. The addition of 200 μL of BCA reagent to 10 μL of sample and standard solutions was followed by incubation at 37°C for 30 minutes. Samples were measured at 595 nm using a microplate spectrophotometer (Biotek ELx800) and data transformed using bovine serum albumin (BSA, Sigma-Aldrich, UK) standard curve. The stability of the substrates was assessed by incubation in sterile deionised water at 1, 7 and 14 days. The experiments were performed in triplicate on different days.

The distribution of the functional groups onto coated substrates was observed after modifying CGen3K(YIGSR)_16_ with fluorescein-5-isothiocyanate (FITC, Sigma-Aldrich, UK). This was prepared by mixing dendrons (0.005 mmol) with a FITC solution (0.025 mmol) and 0.05 mmol DIPEA in 2mL DMF for 8 hours in the dark. The FITC-conjugated CGen3K(YIGSR)_16_ was then washed with DMF and allowed to air-dry before being cleaved and attached to PolyK using the same processes described above. The deposition of FITC-labelled substrates was detected using an inverted microscope equipped with a green wavelength fluorescence filter (Excitation/Emission 494/520 nm, Leica) with a 10x eyepiece. Images were randomly taken for each experimental substrate and qualitatively analysed using TCP and non-modified PolyK substrates treated with FITC as positive and negative control.

The surfaces of coated substrates were observed using a field gun emission scanning electron microscope (FEG-SEM, Zeiss Sigma) after mounting samples on 0.5 aluminium specimen stubs using carbon mounting tabs (Agar Scientific, UK) and coating with 4nm of platinum in a Quorum Q150T ES sputter coater. The SEM images (n = 12) were then analysed for surface roughness and profile parameters including root mean square deviation [Rq] and total peak height [Rt] using an established method denominated the SufCharJ plugin (http://rsb.info.nih.gov/ij) [18].

### hMSC culture conditions

Frozen hMSCs (passage (P) 2, Lonza EU, PT-2501) were re-suspendend in 5 mL of TheraPeak MSCGM-CD serum-free cell culture medium (Lonza EU) and centrifuged at 500 g for 5 minutes. Cells were then counted and plated directly onto TCP, non-modified and modified-PolyK surfaces at seeding density of 7x10^3^ cells/cm^2^. hMSCs were incubated at 37°C and 5% CO_2_ from 4 hours up to 7 days. Their cell morphology was assessed by phase contrast microscopy (Leica DM2500) and SEM as previously described whereas viability by fluorescence microscopy (Inverted Axiovert 25 Zeiss) after staining cells with a Hoescht 33342 Propidium Iodide (HPI, Fisher Scientific, UK) solution.

Cell proliferation studies were performed on hMSCs after being harvested as before and subcultured from passage two to four according to the supplier’s instructions. Briefly, cells were detached from substrates using 0.1% w/v trypsin/EDTA solution for 8 minutes at room temperature and centrifuged at 500g for 5 minutes. hMSCs were re-suspended in 1 mL of medium and repeatedly pipetted to break up any formed aggregate. The samples were then re-plated under the same standard culture conditions for seven days while their medium changed every 3 days. hMSC expansion growth rate was evaluated at each cellular passage and expressed in terms of total cell number (x10^5^) and cumulative population doublings (n = 6). hMSCs were also characterised for MSC-associated markers including CD73 (Abcam UK cat.n. Ab54217), CD105 (Abcam UK cat. n. Ab11414) and CD44 (Abcam UK cat. n. Ab6124) according to a published protocol [19]. The experiments were repeated for n = 9 replicates.

### Morphological analysis

hMSCs adhering onto CGen3K(YIGSR)_16_–modified PolyK substrates and control substrates were photographed using phase contrast microscopy (Leica DM2500) with a x20 objective lens. Where formed, spheroids were measured for their dimensions at each passage using an Image J program and their size values expressed as mean diameter ± standard deviation (n = 12).

Both integrin distribution and clustering were also assessed by an automated method using the “analyse particles” function of Image J software16 (https://imagej.en.softonic.com/). The particle threshold size was set between 1–500 pixels for integrin distribution and 1–10 pixels for integrin clustering, respectively. Data were expressed as mean x standard deviation (n = 9).

### Analysis of marker expression by immunofluorescence

Immunostaining was the technique of choice to test the ability of CGen3K(YIGSR)_16_ to (i) encourage hMSCs’ adhesion via β1-integrin and cell-to-cell interaction N-cadherin, (ii) modulate cytoskeletal organisation, cell migration and hypoxic conditions (i.e. Rho-A, Ab54835, Abcam UK; CXCR4, Ab1670, Abcam UK; and HIF-1α, Ab113642, Abcam UK) and (iii) retain multipotency through stem cell marker regulation (Nanog, Ab80892, Abcam UK; and OCT-4, Ab91194, Abcam UK). Except for Nanog and Oct-4, all primary antibodies were assessed within cells at passage 3 since it is widely accepted to be the passage after which hMSCs start losing their multipotent phenotype [20].

Briefly, the cells on both non-modified and dendron-modified substrates were fixed using chilled methanol for 10 minutes at -20°C and incubated with 1% w/v bovine serum albumin (BSA, Sigma-Aldrich UK) in phosphate buffered saline (PBS, Sigma-Aldrich UK) for 1 hour at room temperature. The samples were then treated with primary anti-human antibodies (1:100) at 4°C overnight before being immunolabelled with goat anti-mouse 488- or 594-conjugated secondary antibodies (1:100, A32723, A-11005, Fisher Scientific UK) in the dark for 1 hour at room temperature and counterstained with DAPI for confocal fluorescence imaging (20x objective lens, Leica TCS SP5).

Cytoskeleton organisation was investigated by staining the actin filaments of hMSCs with rhodamine/phalloidin (1:100 in PBS, Sigma-Aldrich UK) for 1 hour, room temperature and observed using fluorescence microscopy (NIKON Elipse TE2000U) at a 20x eyepiece.

### Statistical analysis

For all experiments, data were expressed as means ± standard deviation (SD) and statistically analysed using ANOVA test. Data were considered significantly different at p ≤ 0.05.

## Results

### Substrate characterisation and analysis

Analytical analysis confirmed by HPLC the successful synthesis of CGen3K(YIGSR)_16_ whereby the degree of purity for the single eluted peak (retention time 16 minutes) was always higher than 91% with a TLC Rf of 0.7. MS results provided the positive identification of the pure dendrons with a peak at 1405 (M+H)^5+^.

The functionalisation of PolyK with CGen3K(YIGSR)_16_ was demonstrated by a colorimetric method for terminal amino groups indicating that the total exposed amino group concentration in CGen3K(YIGSR)_16_ -modified PolyK was higher (2.7 μg/mL) than the non-modified PolyK (1.6 μg/mL).

**Fig 2:**
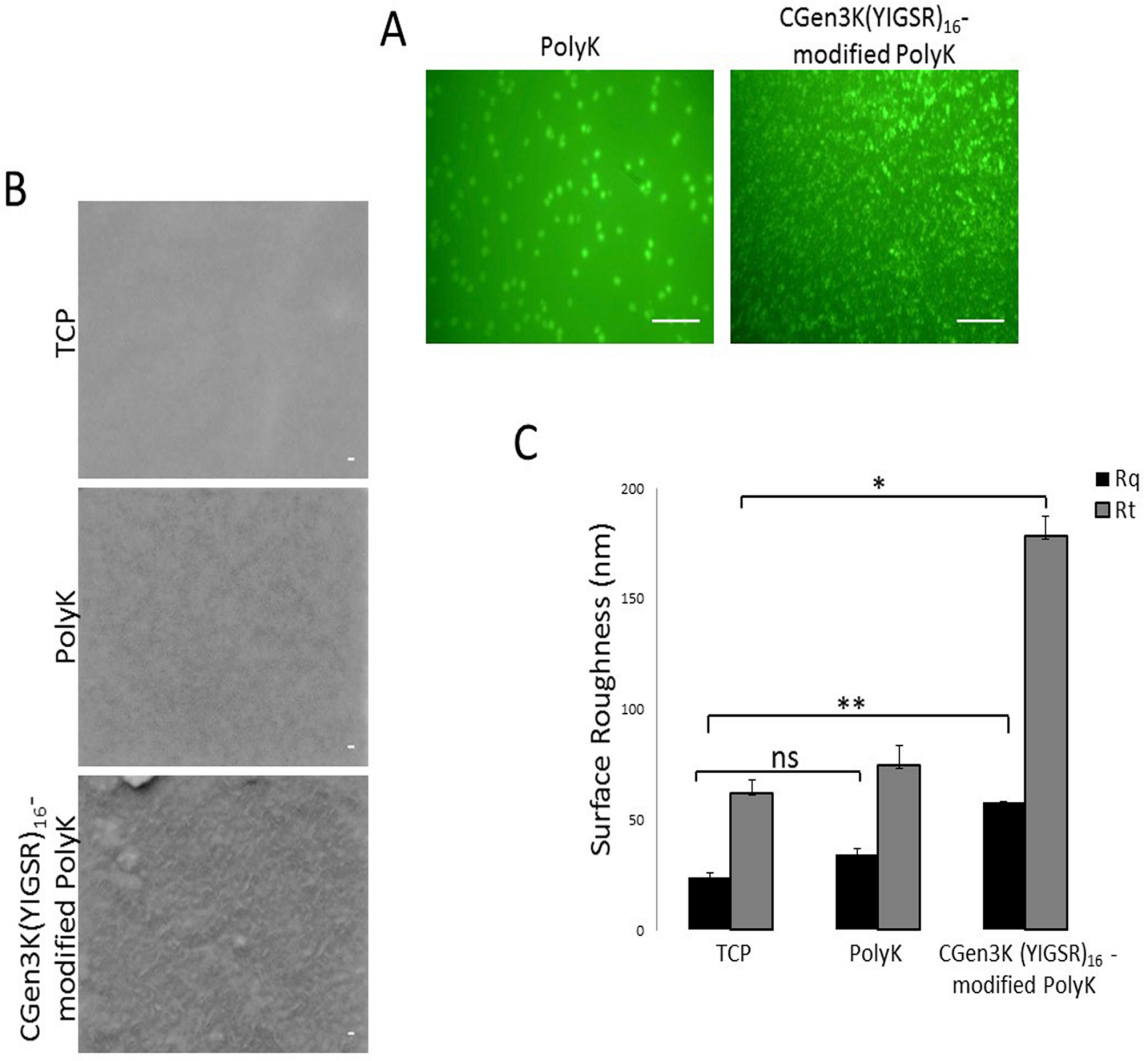
Surface imaging and measurement. The non-modified and CGen3K(YIGSR)_16_- modified PolyK surfaces were assessed by (A) fluorescence microscopy (scale bar = 100 μm), (B) SEM (scale bars = 20 nm) and (C) roughness [Rq = square deviation; Rt = total peak height]. (*P ≤ 0.01, **P ≤ 0.001, ns = no significant; mean ± SD; n = 12).

An *ad hoc* modification of PolyK and CGen3K(YIGSR)_16_-modified PolyK with a FITC fluorescent probe allowed the study of the substrate distribution when deposited on the surface of TCP (Fig 2A). CGen3K(YIGSR)_16_-functionalised PolyK formed dense and distributed clusters of fluorescence (Fig 2A) across the whole surface and maintained stability in aqueous medium over a period of 14 days. The evaluation of exposed primary amino groups was stable when measured in CGen3K(YIGSR)_16_-modified PolyK at 1, 7 and 14 days (79.6±2.11 μg/plate) and higher than non-modified PolyK (17.2±2.7 μg/plate).

SEM micrographs demonstrated that unlike uncoated TCP, both non-modified and dendron-modified PolyK substrates showed charged spots under the electron beam and the appearance of a rough surface (Fig 2B). However, the surfaces of the wells functionalised with CGen3K(YIGSR)_16_, showed a more defined mesh-like topography. These findings were corroborated by the measurement of their Rq and Rt parameters that were found to be higher than those of PolyK and TCP surfaces, respectively (Fig 2C).

### Effects of non-and modified PolyK substrates on hMSC adhesion, organisation and proliferation over passaging

**Fig 3:**
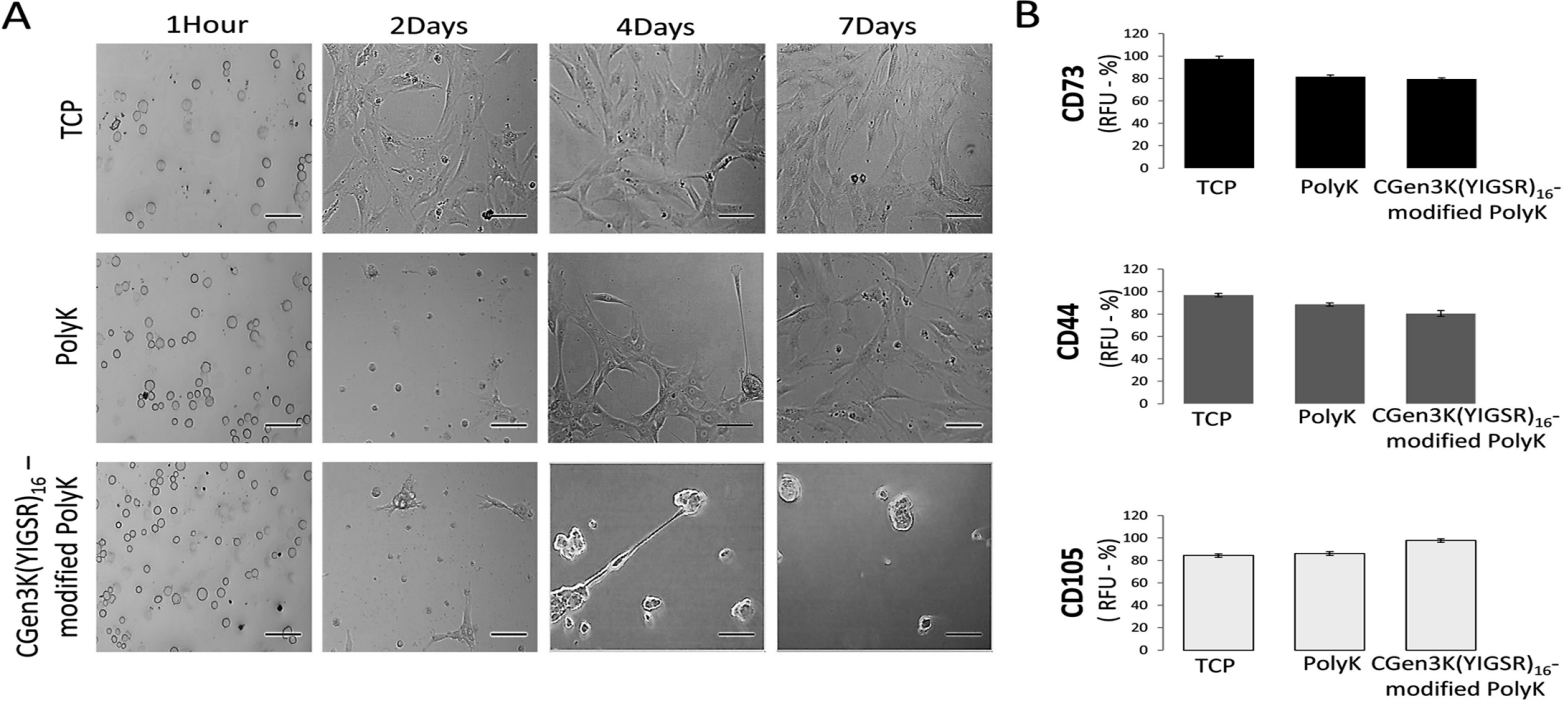
Effects of distinct substrates on hMSC morphology and surface marker regulation at passage 2. (A) Phase contrast microscopy of adhering hMSCs onto TCP, PolyK and CGen3K(YIGSR)_16_-modified PolyK (scale bar = 150 μm), (B) expression of the multipotency markers CD73, CD44 and CD105. (Mean ± SD; n = 9).

**Fig 4:**
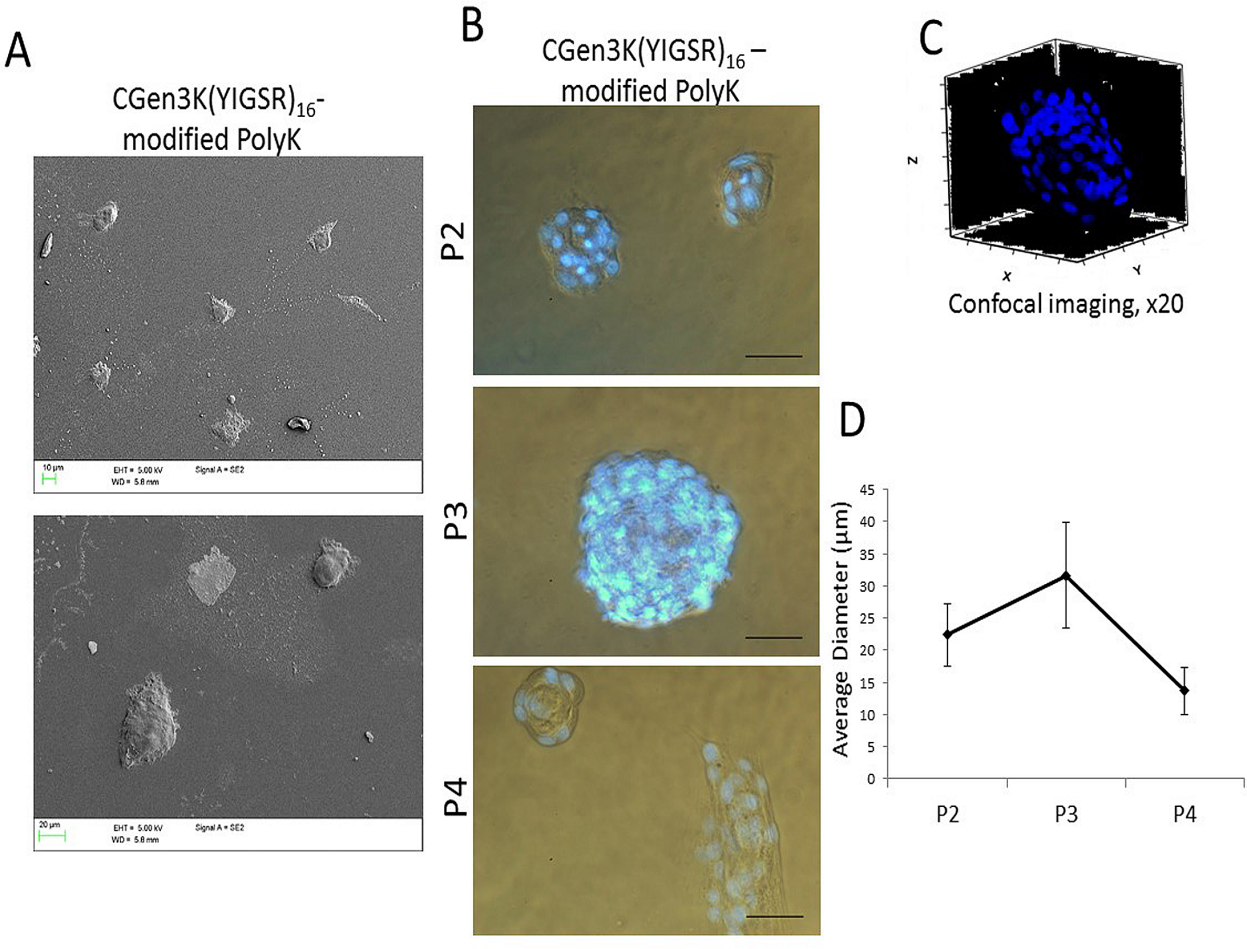
hMSC spheroid formation on CGen3K(YIGSR)_16_-modified PolyK substrates. (A) SEM images showing hMSCs spheroid morphology, (B) cell integrity and viability staining (blue dots; scale bar = 50 μm), (C) typical 3D structure (x20, Z-stacked image of Hoechst 33342 stained nuclei) and (D) comparative analysis of spheroid size at P2, 3 to 4. (Mean ± SD; n = 12).

**Fig 5:**
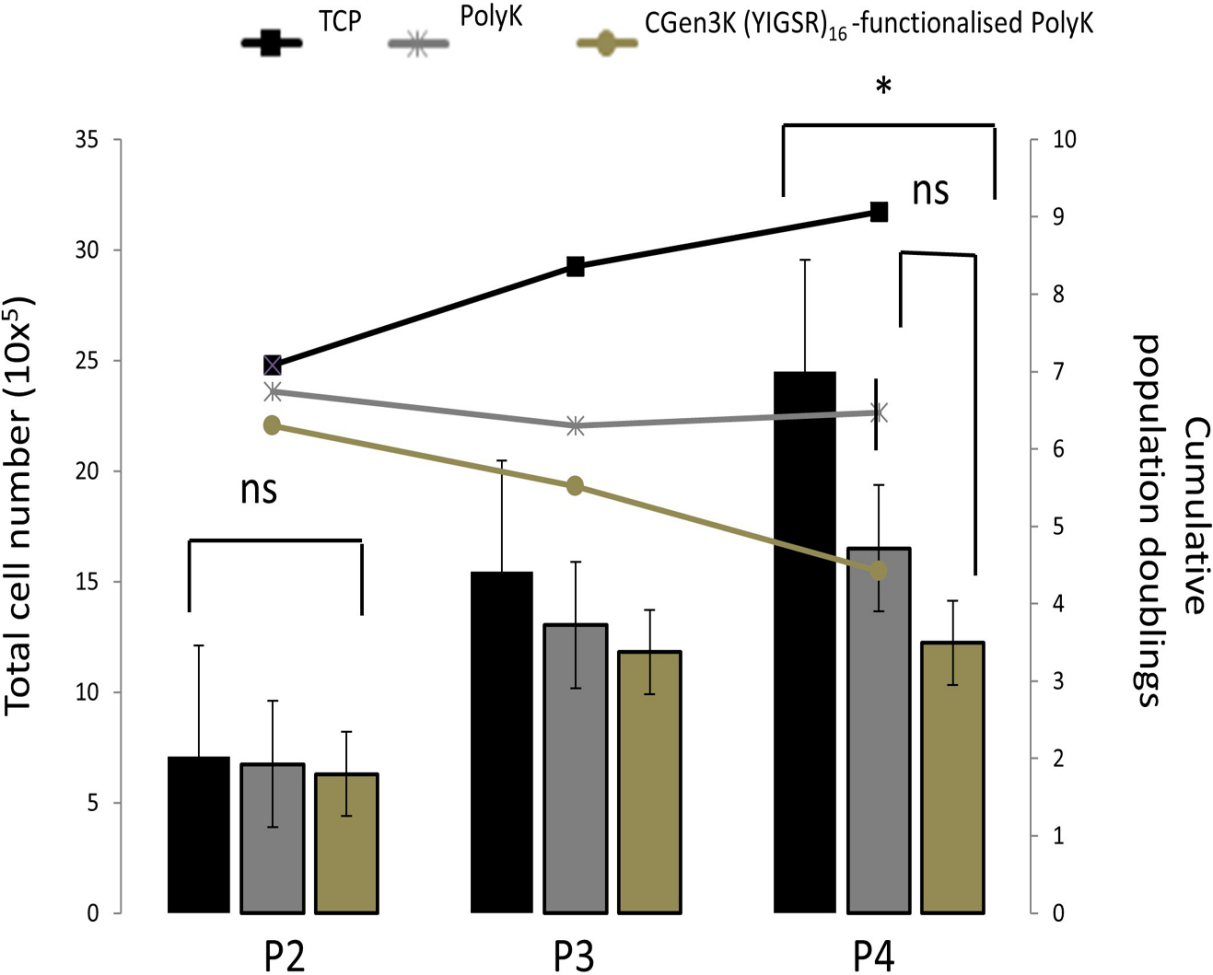
Proliferation of cells when grown onto TCP, non- and CGen3K(YIGSR)_16_-modified PolyK substrates over passaging. Columns report the total number of hMSCs while lines indicate the cumulative population doublings. (*P ≤ 0.01; ns = no significant; mean ± SD; n = 6).

Both non- and CGen3K(YIGSR)_16_-modified PolyK substrates could encourage the adhesion of cells after 4 hours (Fig 3A and S1 Fig). When compared to the control cells, these hMSCs exhibited a round morphology and formed cell aggregates within 2 days of culture. However, cells cultured onto non-treated PolyK substrates became elongated, aligned and closely packed in a manner similar to the morphology observed for cells cultured on TCP. Contrarily, rounded cells organised as multicellular aggregates were still visible on the CGen3K(YIGSR)_16_-modified PolyK substrates by Day 7 incubation. At P2, these cells were defined to be nearly 95% positive for CD73, CD44 and CD105 (Fig 3B) and to form regular and compacted spheroids (Fig 4A) that appeared larger within P3, but elongated and smaller at P4 (Fig 4B) as confirmed by changes in their average size throughout cellular passages (Fig 4C). Interestingly, cells cultured on substrates modified with generation 2 dendrons did not induce spheroid formation and the morphology of the cells was more similar to that found in cells adhering on non-functionalised PolyK showing comparable CD44 localisation (see S2 Fig). However, only TCP surfaces provided higher cell numbers after four passages (Fig 5).

### Laminin dendron substrates induce cytoskeleton re-modelling via β1-integrin and cadherin activation

**Fig 6:**
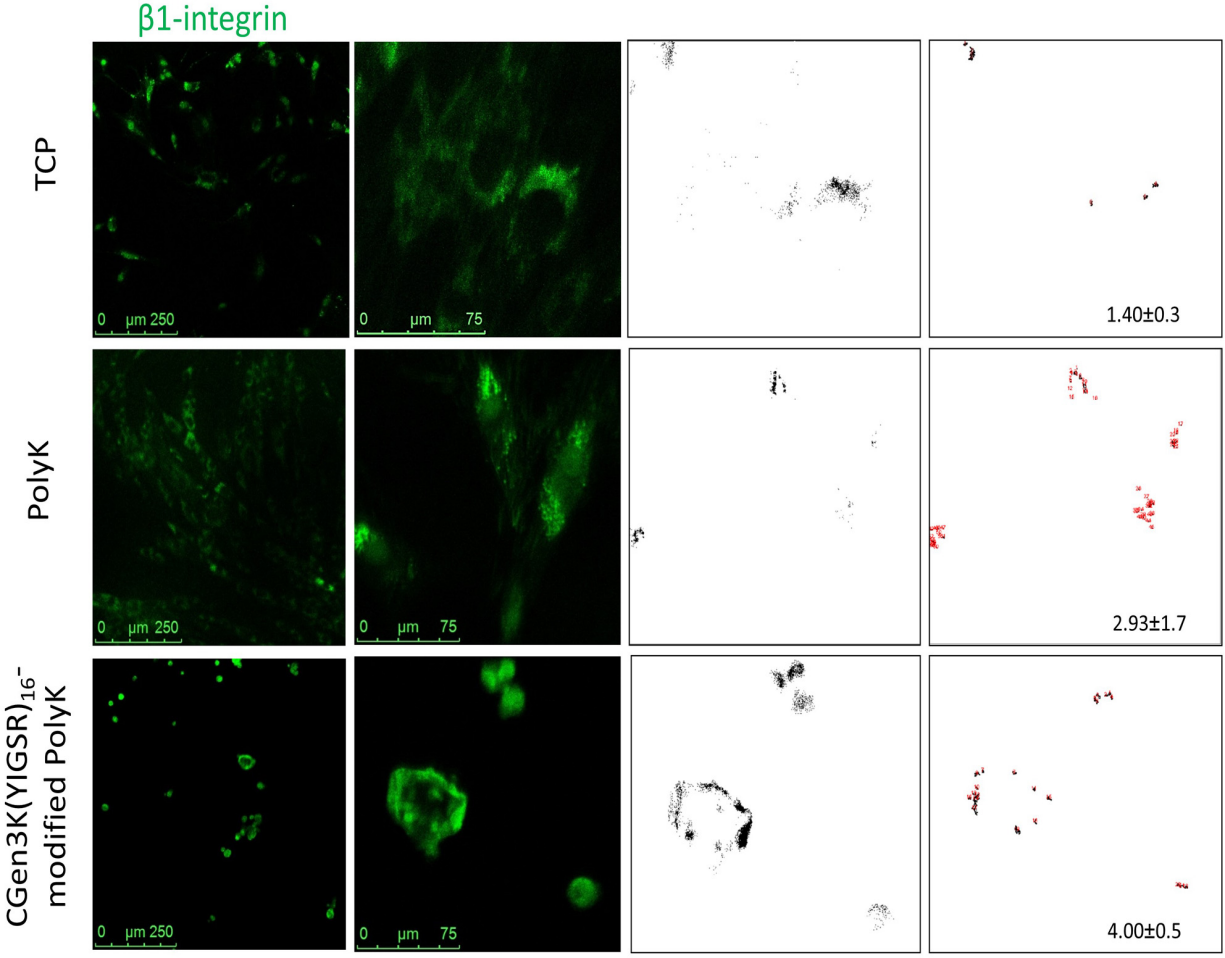
Localisation and clustering of β-1 integrin within hMSCs. Changes in β-1 integrin localisation (green staining) within cells seeded onto distinct substrates (scale bars = 250 and 75μm). The immunostaining was related to the presence of nuclei and distribution (black dots) and clustering (red dots) of integrin at P3. (Mean ± SD; n = 9).

**Fig 7:**
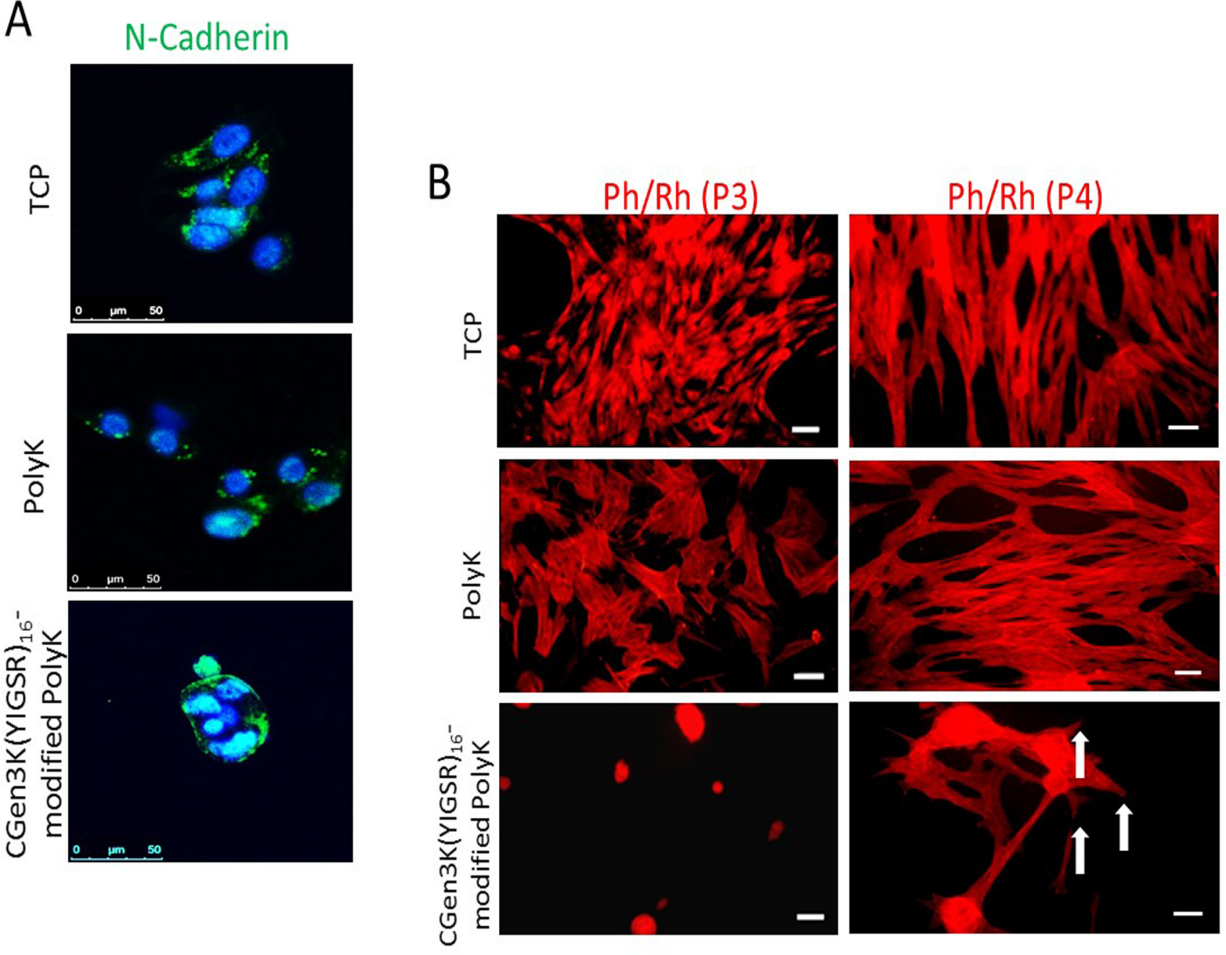
Expression and localization of N-cadherin and changes in cytoskeleton organisation within cells. (A) Distribution of N-Cadherin (green staining) in relation to cell nuclei (blue) at P3 (scale bar = 50 μm), (B) cytoskeleton organisation on the different substrates. AT P4, hMSCs adhering on CGen3K(YIGSR)_16_-modified PolyK showed a tendency to form a cytoskeleton consisting of immature filaments (white arrows). (Scale bar = 50 μm).

These interesting results were tightly linked to the ability of the CGen3K(YIGSR)_16_ to control cytoskeleton formation by regulating integrin and cadherin localisation. As shown in Fig 6, cells cultured onto all tested substrates were positive for β1-integrins. Specifically, the integrin staining appeared as punctuate patterns localised around the nucleus and throughout area of hMSCs when cultured on both TCP and PolyK substrates. β1-integrins were only detected at the periphery of the multicellular spheroids when hMSCs were seeded on dendron-modified PolyK. In addition, both receptor distribution and clustering were found to be higher in CGen3K(YIGSR)_16_ [4.00±0.5 pixel/cell] than the PolyK [2.93±1.7 pixel/cell] and TCP [1.40±0.32 pixel/cell], respectively. Similar trend was observed for N-cadherin whereby this marker remained confined to the cells located at the periphery of the spheroids adhering onto CGen3K(YIGSR)_16_-modified PolyK and only detected in the perinuclear region of cytoplasm within the spread morphology of hMCSs adhering on TCP and PolyK substrates (Fig 7A). Such alterations in integrin and cadherin localisation were related to changes in cell shape and cytoskeleton organisation (Fig 7B). hMSCs onto TCP assumed the familiar flat, spindle-shaped morphology of fibroblast-like cells with well-developed F-actin fibres from passage (P) 3 to 4. Similarly, the cytoskeleton appeared well organised within cells-adhering PolyK substrates, but their actin fibres were initially less pronounced than those on TCP. Noticeably, hMSCs grown on CGen3K(YIGSR)_16_ formed regular spheroids that showed no actin stressed filaments. However, hMSCs gradually dissociated into single cell which exhibited a fibroblast-like morphology and discontinuous actin cytoskeleton as observed at P4 (Fig 7B, white arrows).

### Changed localisation of Rho-A enhances regulation of CXCR4 and HIF-1α within spheroids-forming hMSCs

To gain insight into the functional mechanisms triggered by laminin dendron substrates, Rho-A family GTPases has been investigated as known to be a key pathway for the activation of cytoskeleton organisation during cell migration and differentiation [21].

**Fig 8:**
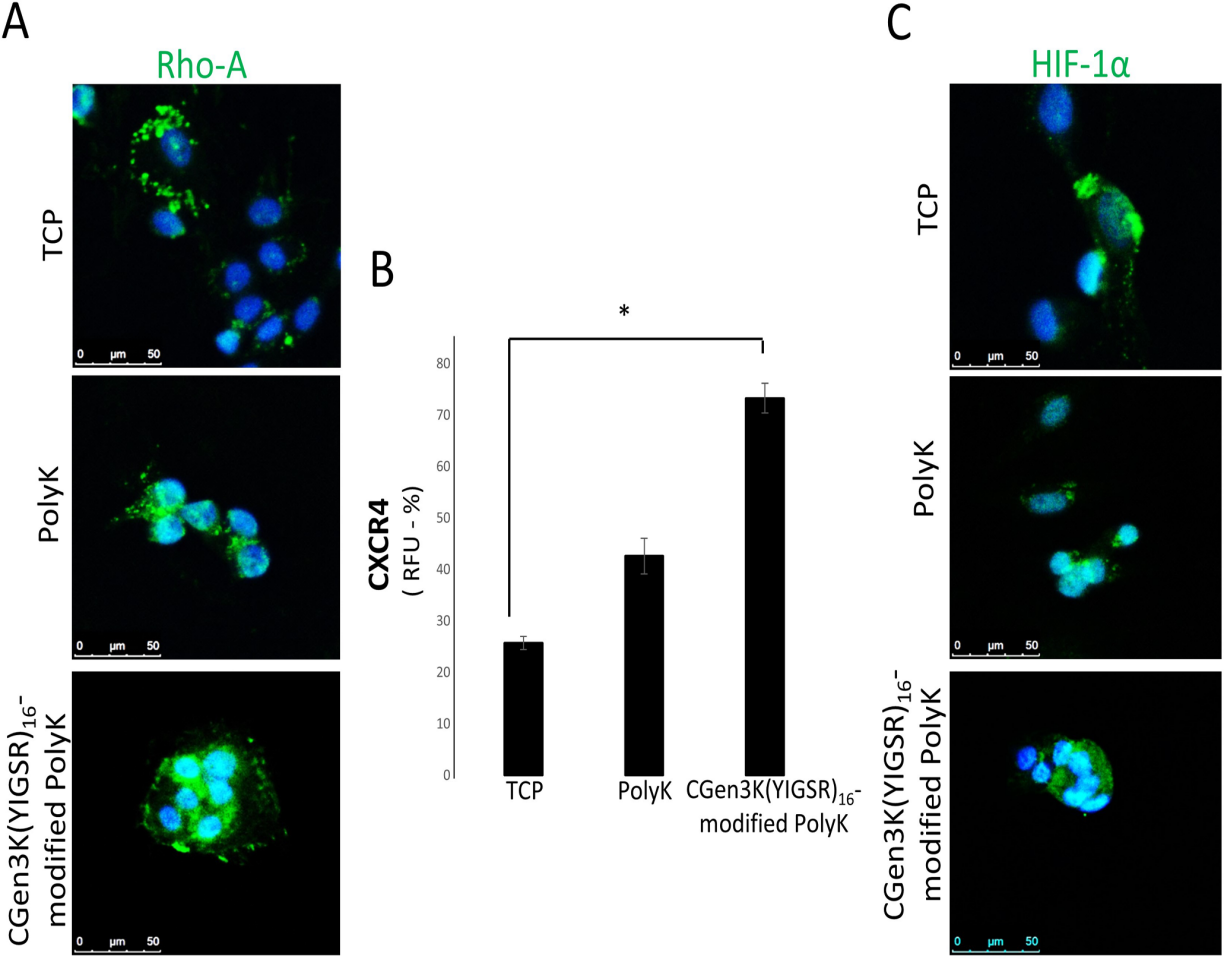
Characterisation of hMSC markers in relation to cytoskeleton activation and cellular migration. hMSCs were stained for (A) Rho-A (marker of cytoskeleton inhibition pathway, green staining) and (B) CXCR4 (marker of migration). Rho-A localisation was assessed in relation to cell nuclei (blue staining) while CXCR4 was quantified by measuring the relative fluorescence unit (RFU) of the immunostaining (*P ≤ 0.01; mean ± SD; n = 6). (C) Localisation of HIF-1α (immunostaining = green; nuclei = blue) was evaluated within cells at P3 (scale bar = 50 μm).

Interestingly, a considerable expression of Rho-A protein was found in all the cells and at the leading edge of the spheroids. This pattern was no longer detected or only vaguely observed throughout the cytoplasm of hMSCs adhering on TCP and it was uniformly, but weakly expressed around the nuclei of hMSCs adhering onto non-modified PolyK substrates (Fig 8A). The changed distribution of Rho-A within hMSCs has been associated with a reduced response of CXCR4 within control cells where levels of its relative fluorescence unit (RFU) had an approximately twofold decrease when compared to spheroid-forming hMSCs on the dendron-modified substrates (Fig 8B). Cells cultured on dendron-modified substrates expressed high levels of HIF-1α which was mainly localised in the nuclear areas. This was found to be either undetectable or distributed in the perinuclear of cells growing as monolayers on TCP and PolyK (Fig 8C).

### CGen3K(YIGSR)_16_-modified PolyK regulate Nanog and Oct-4 and reinforce stem cell pluripotency

**Fig 9:**
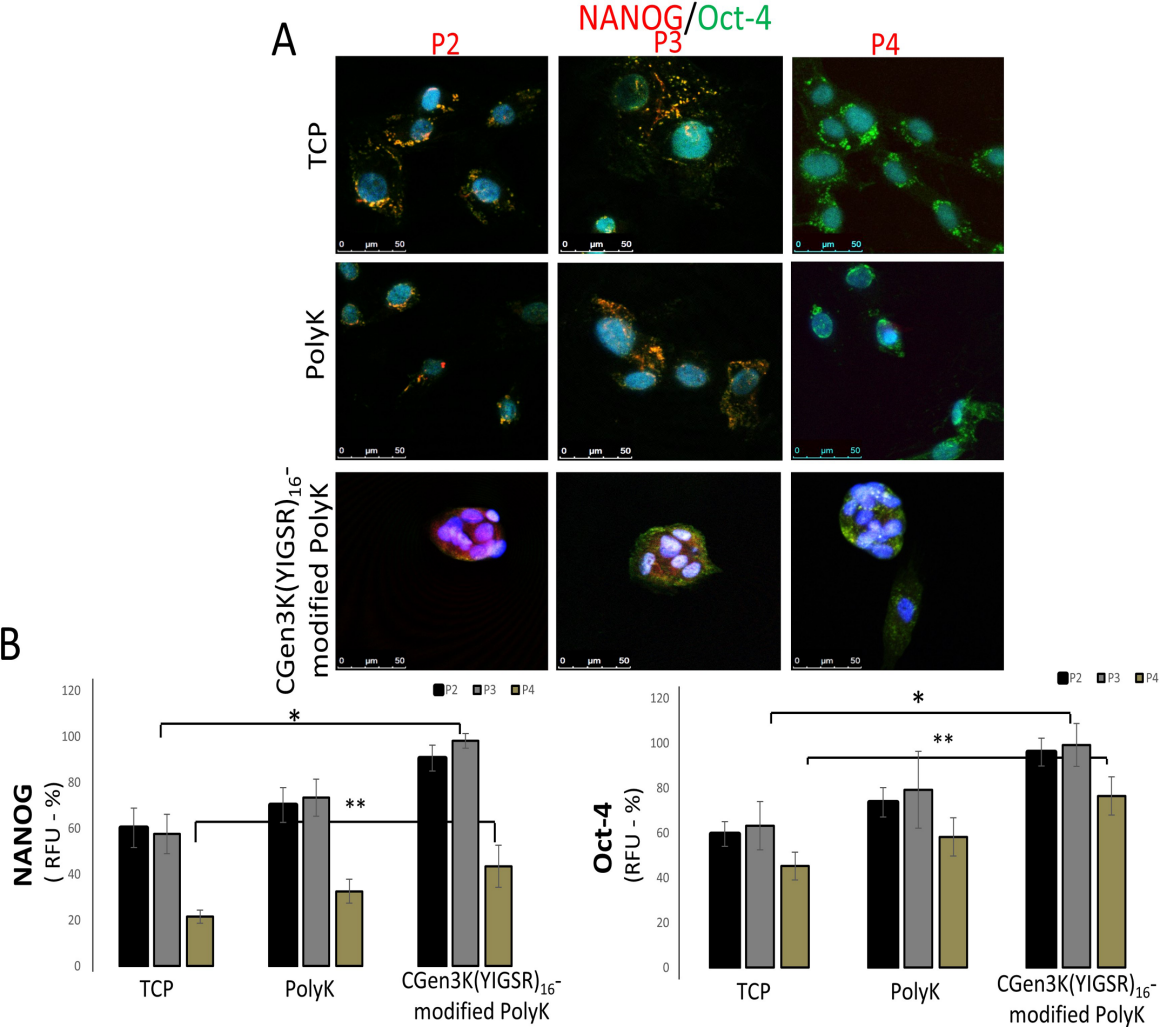
Expression and localisation of Nanog and Oct-4 within hMSCs seeded onto TCP, PolyK and CGen3K(YIGSR)_16_-modified PolyK over passaging. hMSC pluripotency was investigated by staining cells with Nanog (red immunostaining) and Oct-4 (green immunostaining). (Co-localisation of Nanog/Oct-4 appeared as purple staining). Their distribution was assessed in relation to cell nuclei (blue staining) and imaged by confocal microscopy (scale bar = 50 μm). (B) Levels of marker expression were related to the RFU of cells at P2, 3 and 4. (*P ≤ 0.01; **P ≤ 0.001; mean ± SD; n = 6).

As it is recognised that Nanog and Oct-4 regulate potency and self-renewal in embryonic stem cells [22] and more recently found expressed in hMSC [23], these two stem cell markers were investigated in relation both to the process of spheroid formation and to passaging. In this work, hMSCs on TCP and PolyK showed perinuclear-cytoplasm distribution of Nanog protein which strongly overlapped with the localisation of Oct-4 at P2 (Fig 9A). The expression of these MSC markers was found to be either null (Nanog) and/or mainly distributed in the cytoplasm (Oct-4) of cells seeded on control substrates upon passaging. Contrarily, Oct-4 and Nanog proteins were co-localised with the nuclei of spheroid-forming cells onto dendron-exposing surfaces. However, the dissociation of cells from spheroids observed at P4 on the dendron-modified substrates induced the re-distribution of both markers from the nuclear region to the perinuclear-cytoplasm. Thus, the RFU of both Nanog and Oct-4 was found distinct when analysed within cells cultured onto TCP, non-modified and dendron- modified PolyK substrates from P2 to P4 (Fig 9B). Throughout P2 to 3, the RFU of both Nanog and Oct-4 was significantly greater in cells onto CGen3K(YIGSR)_16_-modified PolyK, but RFU of Nanog sharply declined to similar levels of those observed in monolayer cells seeded onto TCP and PolyK compared to Oct-4 which remained higher within spheroid-forming hMSCs at P4.

## Discussion/Conclusions

*In vitro*, hMSCs form a heterogeneous population of cells mainly containing spindle-shaped and flattened cells [24]. Currently, there is no standard culture method that is widely accepted for pre-clinical handling purposes of hMSCs and it is known that variations in the culture methodologies can affect their native characteristics and self-renewal properties [25].

In this study, a new type of biomimetic substrate with the potential to control hMSC pluripotent phenotype was developed to sustain serum-free culturing. Hyperbranched poly(ϵ-lysine) dendrons were designed to expose at their uppermost branching generation YIGSR, the laminin sequence recognised to mediate stem cell adhesion in the stem cell niche [26]. These dendrons were grafted to the linear PolyK, a well-known substrate for cell culturing [27]. The combination of a mesh-like topography, similar that of the collagen type IV with the ordered presentation of the laminin-specific sequence, was deemed appropriate to mimic the ultrastructure and main biochemical features of the BM in single synthetic substrates.

Modifications of PolyK and poly-(ethylene glycol) (PEG) with specific ECM proteins and short peptides like YIGSR and RGD have previously been proposed to achieve better control over cell phenotype [28]. However, one crucial limitation of this approach was the stability of these substrates in culture conditions. Lack of nanometric topography and ligand-presentation control have been shown to encourage stem cell adhesion rather than favouring their long-term proliferation in an undifferentiated state [29, 30]. Here, the chemical functionalisation of PolyK with CGen3K(YIGSR)_16_ significantly increased the substrate stability over a period of 14 days of incubation. These interactions allowed the homogenous coating of the well surface (e.g. TCP) by revealing a complex matrix of intertwined fibres and nodes similar to those observed in the BM ultrastructure of the native stem cell niche. Accordingly, the nano-topography of this type of substrate and its ability to expose spatially-ordered bioligands (YIGSR) appear to be key features for the formation of hMSC spheroids like those found in the natural niches [31]. Previous works have demonstrated the effect of substrate topography as well as of bioligand spacing and distribution on hMSC morphology and phenotype [32]. They have been developed to ensure the spacing of bioligands responsible for cell adhesion through integrin binding [33, 34]. For example, using nano-lithography and nanoimprinting techniques, RGD sequences have been orderly distributed at nanoscale level (60–70 nm) on surfaces showing the effect of surface topography on cell spreading [35, 36]. These studies reported the need for clusters made of a minimum of four integrins as a condition necessary for the formation of focal adhesion points in the cell. Dalby *et al*. 2007 [37] and McMurray *et al*. 2011 [38] adopted electron beam lithography and hot embossing to control the distribution of nanopits in poly (methyl methacrylate) and polycaprolactone substrates showing an improvement in the retention of the hMSC multipotent phenotype [39, 40]. Thus, the control of MSC fate has been shown to depend on the morphology that the cell acquires when adhering on different topographies and spacing of key bioligands [41]. McBeath *et al*. 2004 [42] have demonstrated that when hMSCs are forced to adhere on extracellular matrix islands they tend to become round-shaped and differentiate into adipocytes as opposed to spread cells that get an osteoblast phenotype. The link between nano-topography, bioligand density/patterning and hMSC behaviour is also confirmed by this work. hMSCs adhered onto substrates of Gen3K dendrons aggregated as spheroids rather than becoming spindle-shaped fibroblast-like cells as observed onto substrates of both Gen2K dendrons and un-modified PolyK. This implies that only Gen3K(YIGSR)_16_ were able to allow facile tethering of YIGSR (e.g. YIGSR in Gen2 = 8; Gen3 = 16) which in turn mediated the formation of spheroids through activation of integrin and N-cadherin receptors. These cell adhesion proteins were found to be co-localised at the periphery of the spheroids likely due to an improved integrin clustering which is known to alter the spatial distribution of N-cadherin within focal adhesions [43]. The integrin-dependent control of N-cadherin activity has recently been revealed to also mediate cell adhesion, spreading and migration by regulating intracellular signalling capable to mediate formation of well-defined focal adhesion complexes at points where actin filaments are developed [44]. More specifically, integrin clustering leads to the activation of the Rho-GTPases pathway inducing rapid morphological changes that are accompanied by disruption of stress fibres and disappearance of focal adhesion within cells [45, 46]. Consistent with this finding, hMSCs at the edge of the spheroids promoted accumulation of F-actin filaments while modulated the assembly of integrin-dependent focal adhesions. After attaching and spreading onto the dendron-modified substrates, these cells appeared to retract their pseudopodia filaments and form multicellular spheroids as confirmed by micrograph images. CGen3K(YIGSR)_16_- modified PolyK substrates may have exerted a physical force on hMSCs by altering actin activity in their cytoskeleton thus leading to changes in cell shape and intracellular signalling. These appeared to be associated with N-cadherin-dependent cell-cell contacts which were up-regulated in migrating cells observed at the leading edge of spheroids. Conversely, hMSCs inside the spheroids maintained reduced N-cadherin-mediated interactions, but revealed overexpression and activation of Rho-A protein since the very early stage. However, reduced levels of Rho-A and indeed spheroid sizes were observed from P3 to P4 where increased generation of fibroblast-like hMSCs relative to cell spreading and migration was also detected to take place like on the control plates. It may be speculated that the damage of the integrin receptors generated by the trypsinisation of the cells upon passaging may have abridged their ability to be influenced by the precise topography and bioligand presentation of the dendron-modified substrates. Whatever the mechanisms involved, the results of this work indicate that N-cadherin activity by integrin-dependent signals likely affected cell aggregation via Rho-A regulation during a sensitive time window that corresponded to the changes in hMSC shape from rounded to elongated. The modulated regulation of these molecules might also be influenced by different migratory behaviour of the cells which is consistent with previous works that revealed multiple signalling such as GTPases, to be regulated via CXCR4 [47]. It has been reported that loss of Rho-A activity is associated with increased cell migration due to the combined disruption of integrin and CXCR4 regulation [48]. Nevertheless, due to the proteolytic activity of trypsin, hMSCs adhering on laminin dendron substrates proliferated relatively more slowly when compared to those cultured onto TCP and PolyK surfaces. At P2, the size of each spheroid was relatively smaller than that observed in spheroids undergoing a passage over the same substrate (P3); these gradually reduced diameter and disaggregated into single cells of spindle-like morphology throughout P4. However, hMSCs onto CGen3K(YIGSR)_16_-modified PolyK expressed higher levels of both Nanog and Oct-4 showing hence an enhanced self-renewing pluripotent state maybe favoured by their co-localisation at early passages. The distribution of these markers was initially identified in the nuclei of spheroid-forming hMSCs when high transcriptional activity has previously been demonstrated [49, 50]. The translation of both Nanog and Oct-4 seems to be a very rapid process so that slight alterations (e.g. cell expansion) may induce their downregulation and indeed MSC differentiation into fibroblast-like cells [51]. This work supports these findings, but also reveals an intricate relationship between regulation and distribution of both Nanog and Oct-4 within cells. At P4, hMSCs onto CGen3K(YIGSR)_16_-modified PolyK substrates showed reduced Nanog levels and determined the regulation of Oct-4, mainly observed in the cytoplasm, which is typical of senescence-like alterations including irreversible growth arrest and marked impairment in migratory and homing capability [52].

At the same time, the maintenance of stemness by the cells could also be controlled by the relatively high expression of HIF1α which is known to regulate stem cell pluripotency as well as their proliferation in the bone marrow stem cell niche [53, 54].

In conclusion, the present paper shows that CGen3K(YIGSR)_16_-modified PolyK substrates, synthesised at purity (>95%) and quantities (500 mg/batch) necessary for hMSC culturing in highly reproducible conditions, have the potential of mimicking prime aspects of the microenvironment of an adult stem cell niche. The nano-topography of this type of substrate and its ability to expose the fine spatial presentation of bioligands appear to be key features for the formation of the hMSC spheroids suggesting that, beyond their use for *in vitro* studies, these biomimetic biomaterials could also be used as stem cell substrates in tissue engineering constructs.

## Supporting information

S1 Fig Effects of substrate materials on stem adherent cells at passage 2. Light micrograph images of hMSCs (scale bar = 150 μm).
(TIF)

S2 Fig Morphology and CD44 localisation within hMSCs grown on TCP, PolyK and CGen2K(YIGSR)_8_-modified PolyK substrates. The morphology of hMSCs was observed by (A) phase contrast microscopy (scale bar = 50 μm) and (B) confocal microscopy where the distribution of CD44 marker (green immunostaining) was assessed in relation to cell nuclei (blue staining) (scale bar = 100 μm).
(TIF)
