# Supplementary figures and images for: Hyperbranched poly(ϵ-lysine) substrate presenting the laminin sequence YIGSR induces the formation of spheroids in adult bone marrow stem cells
Source: PLoS One. 2017 Dec 12;12(12):e0187182. doi: 10.1371/journal.pone.0187182 (PMC5726715; doi:10.1371/journal.pone.0187182)

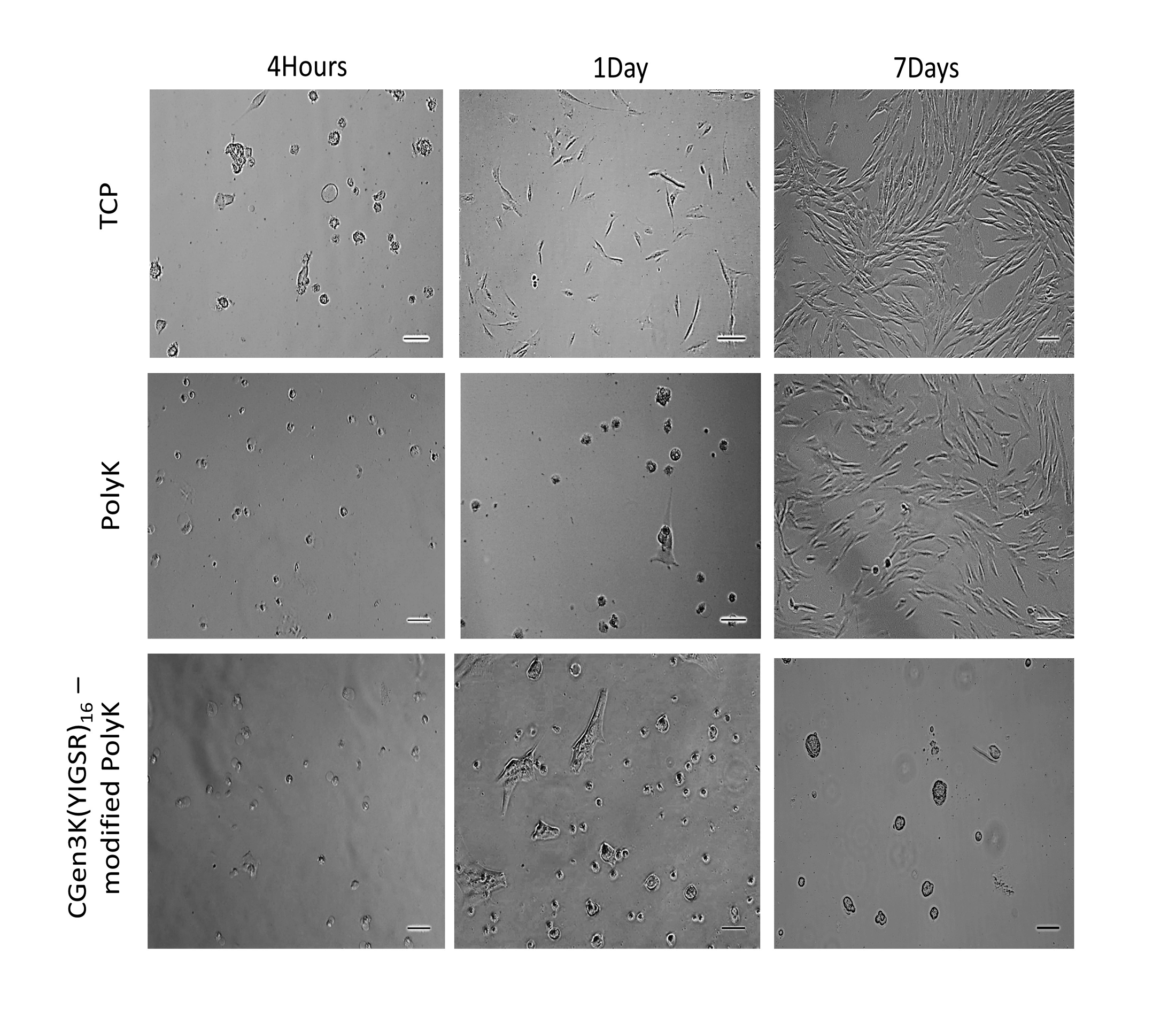

Supplement: S1 Fig — Light micrograph images of hMSCs (scale bar = 150 μm). (TIF) [file pone.0187182.s001.tif]

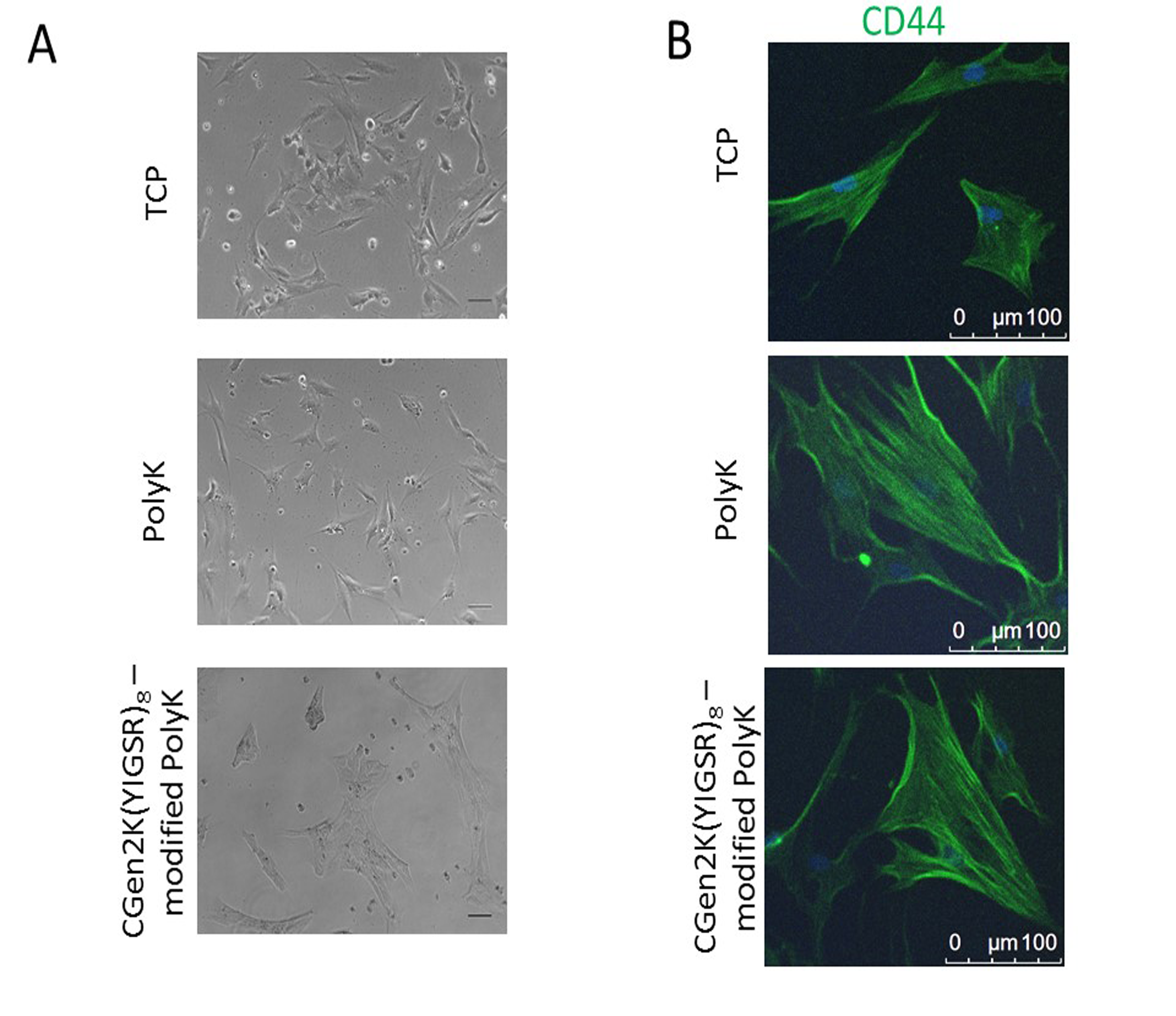

Supplement: S2 Fig — The morphology of hMSCs was observed by (A) phase contrast microscopy (scale bar = 50 μm) and (B) confocal microscopy where the distribution of CD44 marker (green immunostaining) was assessed in relation to cell nuclei (blue staining) (scale bar = 100 μm). (TIF) [file pone.0187182.s002.tif]
